# Congenital hemangioma in spondylocostal dysostosis: a novel association*

**DOI:** 10.1590/abd1806-4841.20164497

**Published:** 2016

**Authors:** Victor Michael Salinas-Torres

**Affiliations:** 1 Hospital General de Tijuana, Instituto de Servicios de Salud Pública en el Estado de Baja California – Baja California, México

**Keywords:** Congenital Abnormalities, Hemangioma, Meningomyelocele, Arnold-Chiari Malformation, Spine, Sternocostal Joints

## Abstract

Congenital hemangioma is a benign tumor caused by dysfunction in embryogenesis and vasculogenesis, which progresses during fetal life to manifest as fully developed at birth. Although hemangiomas are the most common tumor of infancy, rapidly involuting congenital hemangioma has not been described in spondylocostal dysostosis. I report the novel association of congenital hemangioma and spondylocostal dysostosis in a Mexican newborn female patient with neural tube defects. Given the embryological relationship between skin and nervous system, I surmise that this association is not coincidental. I also propose that these morphologic alterations be incorporated to the spondylocostal dysostosis phenotype and specifically looked for in other affected children, in order to provide appropriate medical management and genetic counseling.

## INTRODUCTION

Spondylocostal dysostoses (SCD) are a genetic heterogeneous group of rare disorders characterized by multiple vertebral segmentation defects and rib abnomalities.^1,2^ Although several associated anomalies have been included in the clinical spectrum of SCD, congenital hemangioma has not been documented.^3,4^ Here, I report a previously undescribed case of a rapidly involuting congenital hemangioma in SCD along with neural tube defects.

## CASE REPORT

**Figure 1:**
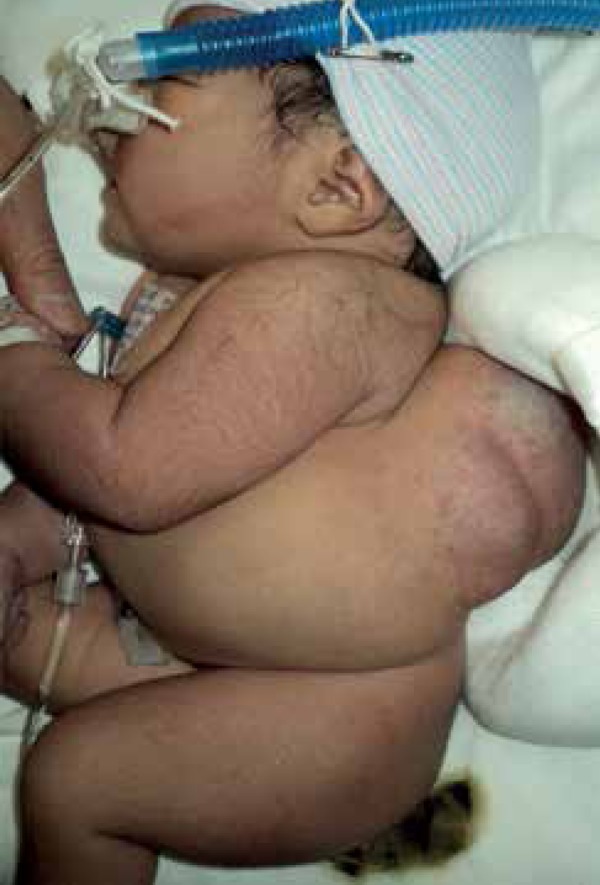
Patient at birth with a thoracolumbar rapidly involuting congenital hemangioma characterized by a raised greyish tumor surrounded by a pale halo with multiple tiny telangiectasias

A Mexican newborn female was the second child of healthy non-consanguineous 24-year-old parents without family history of multiple congenital anomalies/mental retardation (MCA/MR) syndromes. A thoracolumbar meningomyelocele was diagnosed on a prenatal ultrasound. The patient was born at pregnancy week 37 by cesarean section after an uneventful pregnancy (Apgar scores = 4 and 7; birth weight = 3265 g; length = 49 cm; and OFC = 34 cm – all in 50th centile). Clinical examination showed micrognathia, low-set ears, short neck and thorax, abdominal distention. It also revealed a significant thoracolumbar defect (7 x 9 x 4.5cm in size divided by a septum) with a congenital hemangioma. The tumor was greyish in color and characterized by raised edges surrounded by a pale halo with multiple tiny telangiectasias consistent with a rapidly involuting congenital hemangioma (Figure 1). No genital or limb anomalies were observed.

**Figure 2:**
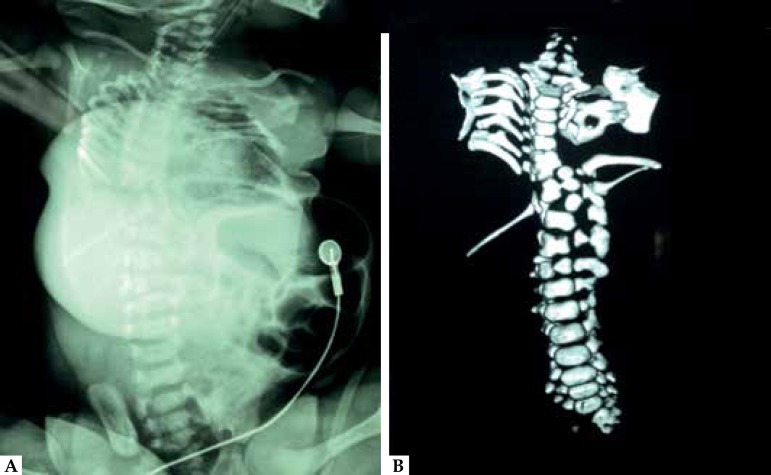
Spinal radiographs **(A)** and 3D computed tomography **(B)** showing narrow thorax, multiple segmentation defects of the vertebrae, malalignment of the ribs with variable points of intercostal fusion, and reduction in rib A B number consistent with SCD

**Figure 3:**
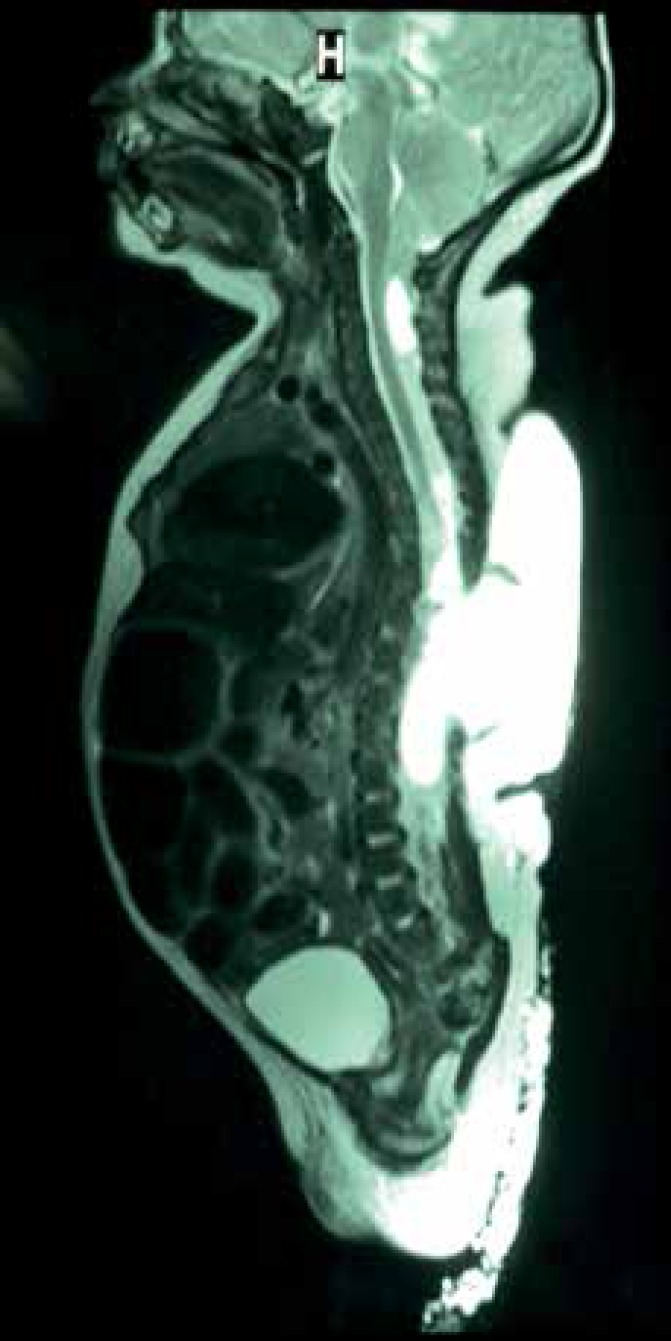
MRI showing cerebellar displacement with tonsillar herniation of 12mm below the foramen magnum consistent with Arnold-Chiari type II malformation, and a thoracolumbar meningomyelocele from T6 to L3

Radiographic findings included: narrow thorax, right hemithorax with incomplete synostosis of posterior costal arches, asymmetry and decreased intercostal space of ribs 1-7, agenesis of ribs 8-9 and 11-12, left hemithorax with asymmetry of ribs 1-2, asymmetry and decreased intercostal space of ribs 3-5, incomplete synostosis of posterior costal arches of ribs 4-5, significant increase in the intercostal space between ribs 5-6, and agenesis of ribs 8-12. Moreover, T7-T8 block vertebrae, T9-T10 hemivertebrae, irregular morphology of T6 and T11-T12, L1 hemivertebra, and thoracolumbar scoliosis were detected (Figure 2). Altogether, these findings were compatible with a diagnosis of SCD. A MRI revealed cerebellar displacement with tonsillar herniation of 12mm below the foramen magnum consistent with Arnold-Chiari type II malformation, and a thoracolumbar meningomyelocele from T6 to L3 with septate cystic lesions (Figure 3). Cardiac and abdominal ultrasonography, as well as ophthalmological examination and G-banded karyotype (> 550 bands) were normal. The patient died four days later due to respiratory failure. The parents did not agree to a skin biopsy nor an autopsy.

## DISCUSSION

The patient's main clinical and radiographic findings – namely narrow thorax, multiple segmentation defects of the vertebrae, malalignment of the ribs with variable points of intercostal fusion, and reduction in rib number – enabled the diagnosis of SCD with the additional features of Arnold-Chiari type II malformation, thoracolumbar meningomyelocele, and a rapidly involuting congenital hemangioma. Associated anomalies in SCD include neural tube defects, congenital heart disease, urogenital and anal anomalies, limb abnormalities, and diaphragmatic, umbilical, and inguinal hernias.^3,4^ However, congenital hemangioma has not been reported yet. Thus, the current observation expands the SCD phenotype and further illustrates the variable expressivity of this disorder.

Although skin and nervous system share a common ectodermal origin, between the third and fifth week of gestation the neural ectoderm separates from the cutaneous ectoderm (disjunction). It is worth observing that this is one of the most vulnerable stages in human development. With a complete separation of neural and cutaneous ectoderms, mesoderm inserts between these two layers to form the meninges, vertebral column, and muscles. In contrast, an incomplete separation results in abnormal development of the spinal cord with or without a persistent connection with the overlying skin, which may also produce abnormalities in the tissues derived from mesoderm and cutaneous ectoderm.^5^ Therefore, the association of SCD with neural tube defects and congenital hemangioma may not be coincidental.

Congenital hemangiomas are benign localized tumors caused by dysfunction in embryogenesis and vasculogenesis, which grow by endothelial cell hiperplasia and progresses during fetal life to manifest as fully developed at birth. Rapidly involuting congenital hemangioma occurs most frequently in the head and neck regions with a complete regression before the age of 14 months. These lesions appears as red-purple plaques with coarse telangiectasia, or as flat violaceous lesions, or as a raised greyish tumor surrounded by a pale halo with multiple tiny telangiectasias, as in the present patient.^6,7^ Moreover, it should be stressed that no hemodynamic complication such as congestive heart failure or hydrops was associated with the thoracolumbar rapidly involuting congenital hemangioma, despite these hemangiomas being high-flow lesions. Therefore, the thoracolumbar location appears to display a lower risk of hemodynamic complication.

Based on clinical and radiographic data, spondylothoracic dysostosis and Casamassima-Morton-Nance syndrome were excluded. These disorders differ from SCD by bilateral fusion of all ribs at the costovertebral joints and the presence of "crab-like" thorax.^3,8^ However, it must be noted that Casamassima-Morton-Nance patients have consistent urogenital and anal anomalies with a poor prognosis in addition to spondylocostal dysostosis.^9^ Additionally, costovertebral anomalies are also a significant feature in VACTERL association. However, the lack of other typical defining component features ruled out this disorder.^10^

SCD has been related to mutations in four genes involved in the Notch signaling pathway: *DLL3* (locus 19q13), *MESP2* (locus 15q26.1), *LFNG* (locus 7p22) and *HES7* (locus 17p13.2) cause SCD-1, SCD-2, SCD-3, and SCD-4 respectively. Recessive mutations in these genes account for ~30% of SCD cases.^1,3^ Additionally, an autosomal dominant form SCD-5, caused by mutations in *TBX6* gene (locus 16p11), has been reported.^2,3^ Such a genetic heterogeneity will likely increase once other genes are identified.

In conclusion, the coexistence of a rapidly involuting congenital hemangioma in SCD represents a novel association. Given the embryological relationship between skin and nervous system, I surmise that this association along with neural tube defects is not coincidental. In addition, I propose that these morphologic alterations should be incorporated to the SCD phenotype and specifically looked for in other affected children, in order to provide appropriate medical management and genetic counseling.

## ACKNOWLEDGEMENTS

The author gratefully acknowledges Dr. Horacio Rivera for his critical review of the manuscript. Also thanks to M. Sc. Rafael A. Salinas Torres for his support and artwork.
